# PPARγ Controls Ectopic Adipogenesis and Cross-Talks with Myogenesis During Skeletal Muscle Regeneration

**DOI:** 10.3390/ijms19072044

**Published:** 2018-07-13

**Authors:** Gabriele Dammone, Sonia Karaz, Laura Lukjanenko, Carine Winkler, Federico Sizzano, Guillaume Jacot, Eugenia Migliavacca, Alessio Palini, Béatrice Desvergne, Federica Gilardi, Jerome N. Feige

**Affiliations:** 1 Nestle Institute of Health Sciences, EPFL Innovation Park, Building H, EPFL Campus, 1015 Lausanne, Switzerland; 2 Center for Integrative Genomics, Faculty of Biology and Medicine, University of Lausanne, 1015 Lausanne, Switzerland; beatrice.desvergne@unil.ch (B.D.); Federica.Gilardi@hcuge.ch (F.G.); 3 School of Life Sciences, Ecole Polytechnique Federale de Lausanne (EPFL), 1015 Lausanne, Switzerland

**Keywords:** Skeletal muscle, regeneration, myogenesis, adipogenesis, muscle stem cells, satellite cells, inflammation, *PPARg*

## Abstract

Skeletal muscle is a regenerative tissue which can repair damaged myofibers through the activation of tissue-resident muscle stem cells (MuSCs). Many muscle diseases with impaired regeneration cause excessive adipose tissue accumulation in muscle, alter the myogenic fate of MuSCs, and deregulate the cross-talk between MuSCs and fibro/adipogenic progenitors (FAPs), a bi-potent cell population which supports myogenesis and controls intra-muscular fibrosis and adipocyte formation. In order to better characterize the interaction between adipogenesis and myogenesis, we studied muscle regeneration and MuSC function in whole body *Pparg* null mice generated by epiblast-specific Cre/lox deletion (*Pparg^Δ/Δ^*). We demonstrate that deletion of PPARγ completely abolishes ectopic muscle adipogenesis during regeneration and impairs MuSC expansion and myogenesis after injury. Ex vivo assays revealed that perturbed myogenesis in *Pparg^Δ/Δ^* mice does not primarily result from intrinsic defects of MuSCs or from perturbed myogenic support from FAPs. The immune transition from a pro- to anti-inflammatory MuSC niche during regeneration is perturbed in *Pparg^Δ/Δ^* mice and suggests that PPARγ signaling in macrophages can interact with ectopic adipogenesis and influence muscle regeneration. Altogether, our study demonstrates that a PPARγ-dependent adipogenic response regulates muscle fat infiltration during regeneration and that PPARγ is required for MuSC function and efficient muscle repair.

## 1. Introduction

Skeletal muscle is a highly plastic tissue, which can adapt to different metabolic or physical challenges, such as physical activity, disuse, or injuries. When muscle fibers are damaged as a consequence of intense exercise, trauma, surgery, or genetic diseases, complementary cellular mechanisms allow skeletal muscle to regenerate and recover tissue architecture and functionality. This regenerative capacity is primarily driven by muscle stem cells (MuSCs), also known as satellite cells, which express the transcription factor paired box 7 (Pax7) [1,2]. As a consequence of injury, MuSCs break quiescence and enter the cell cycle, undertake myogenic commitment after expansion, and reconstitute the contractile architecture by forming fusing to damaged fibers or forming de novo myofibers [3,4]. Successful muscle regeneration is tightly dependent on a temporally controlled inflammatory response. A first wave of pro-inflammatory macrophages clears cellular debris [5] and promotes MuSC expansion while inhibiting their premature differentiation [6]. Pro-inflammatory macrophages are then replaced by a second wave of anti-inflammatory and pro-regenerative macrophages, which switches the muscle stem cell niche to a state permissive to fusion and differentiation to allow regeneration to complete [5,6].

Infiltration of fat in skeletal muscle has been reported in various physiological and pathological settings. In particular, intra-muscular adipose tissue (IMAT; [7,8]) accumulates during muscle diseases with impaired regeneration such as sarcopenia, muscular dystrophies, and rotator cuff tears [9,10,11,12,13]. Genetic experiments in mice with depletion of Pax7^+^ MuSCs have demonstrated that MuSC failure and impaired regeneration directly causes ectopic muscle adipogenesis and fibrosis [14,15], and other studies have associated unsuccessful or delayed muscle regeneration with the development of muscle ectopic fat and fibrosis [16,17,18]. Fibro/adipogenic progenitors (FAPs) are specific progenitor cells residing in the stem cell niche that are distinct from MuSCs and can differentiate to adipocytes in vitro and in vivo [3,19,20]. In response to injury, uncommitted FAPs activate and proliferate to support the myogenic capacity of MuSCs [19,20,21,22]. The fate of FAPs is then precisely regulated during regeneration, in particular through a cross-talk with immune cells where tumor necrosis factor α (TNFα) initially promotes FAP amplification and survival during MuSC expansion, and transforming growth factor β1 (TGF-β1) subsequently induces FAP apoptosis in later phases of regeneration to limit uncontrolled expansion of FAPs [23].

We and others have shown that various models of muscle injury induced by intra-muscular injection of cardiotoxin (CTX) or glycerol lead to ectopic adipogenesis in muscle with accumulation of fat containing cells between muscle fibers [24,25,26,27]. Under physiological conditions, this response is transient and cleared during the terminal phases of regeneration. Given this observation, we hypothesized that ectopic adipogenesis could be important for efficient regeneration and set-out to test this possibility by interfering with adipogenesis through genetic modulation of the peroxisome proliferator-activated receptor γ (PPARγ). PPARγ is a nuclear receptor activated by fatty acids and eicosanoids and is the master regulator of the adipogenesis [28,29]. Pre-adipocytes cannot differentiate in the absence of PPARγ, and PPARγ activation during adipocyte differentiation induces several target genes required for cell cycle exit, adipogenic commitment, and triglyceride metabolism and storage in lipid droplets [30,31,32]. During skeletal muscle regeneration, PPARγ is strongly upregulated three days after injury [24], at the time where FAPs expand to support myogenesis. PPARγ is also expressed in macrophages where it indirectly modulates MuSC function and regeneration via a GDF-3 mediated crosstalk in the muscle stem cell niche [33]. Total invalidation of PPARγ in mice is lethal, and genetic studies in mice have largely focused on tissue-specific conditional knock-outs. Deletion of PPARγ from adipose tissue caused lipodystrophy and confirmed the prominent role of PPARγ in adipocyte differentiation and metabolism, while PPARγ deletion from skeletal muscle caused insulin resistance [34,35]. PPARγ is also important in macrophages where it controls inflammation and foam cell formation during atherosclerosis [36,37,38]. Recent work has demonstrated that the lethality of PPARγ knock-out mice results from placental defects that can be overcome by using an epiblast-specific Cre deleter which removes PPARγ in the entire embryo while maintaining its expression in the placenta during pregnancy [39,40]. In the present study, we used this genetic model of whole-body PPARγ deletion to study muscle regeneration. By analyzing histological and molecular readouts in vivo and the cross-talk between FAPs and MuSCs in vitro, we demonstrate that the loss of PPARγ totally blunts ectopic adipogenesis during muscle regeneration and alters MuSC function and regeneration.

## 2. Results

### 2.1. PPARγ Deletion Completely Abolishes Ectopic Adipogenesis During Skeletal Muscle Regeneration

**Figure 1:**
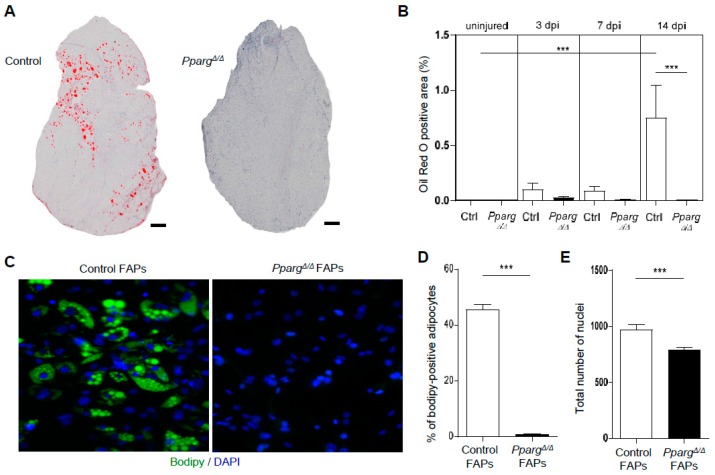
Ectopic adipogenesis during muscle regeneration is abolished in *Pparg^Δ/Δ^* mice. Tibialis anterior muscles from *Pparg^Δ/Δ^* and control littermates mice were injured with 50% glycerol intramuscular injection and collected at 3, 7, and 14 days post injury (dpi), *n* ≥ 7 mice per group. (**A**) Representative image of tibialis anterior (TA) section of control (Ctl) and *Pparg^Δ/Δ^* (KO) mice stained by Oil Red O at 14 dpi. Scale bars: 200 µm. (**B**) Quantification of the area covered by Oil Red O in uninjured muscle and at 3, 7, and 14 dpi in control and *Pparg^Δ/Δ^*. (**C**–**E**) FAPs were isolated from control and *Pparg^Δ/Δ^* muscle by flow cytometry and cultured in adipogenic conditions, with n ≥ 24 cell culture replicates pooled from at least three mice per genotype and replicated twice on different days. (**C**) Representative staining of lipid droplets by bodipy (green) and DNA (blue). Magnification: 20× objective. (**D**) Percentage of differentiated Bodipy-positive FAPs quantified by high content imaging. (**E**) Total number of FAPs quantified by high content imaging via the DAPI staining. *** *p* < 0.001 by one-way analysis of variance (ANOVA) followed by Bonferroni’s test (**A**) and by Mann–Whitney test (**D**,**E**).

Several studies have reported an upregulation of PPARγ during skeletal muscle regeneration, which precedes the infiltration of lipid-droplet positive cells [24,26]. One open question is whether the induction of PPARγ is the causal mechanism leading to fat infiltration, and whether this infiltration is triggered by the adipogenic differentiation of progenitors in mature adipocytes. In order to answer the question, we used whole-body PPARγ null mice (*Pparg^Δ/Δ^* mice) which were recently generated by epiblast-specific Cre deletion which deletes PPARγ in the entire embryo but not in the placenta [40]. Using this strategy, *Pparg^Δ/Δ^* mice are viable and totally lack mature adipocytes throughout the body [40], but have a compensatory fat accumulation in non-adipose tissues leading to increased body weight (Figure S1A). Muscle weight is also lower in *Pparg^Δ/Δ^* mice than in control littermates (Figure S1B), most likely because of impaired metabolic homeostasis and steatosis in these mice, as these conditions have been shown to alter insulin and anabolic signaling in other settings [41,42]. Tibialis anterior muscle of *Pparg^Δ/Δ^* mice and control littermates was injected intramuscularly with glycerol to cause fatty muscle degeneration as previously reported [19,20,24,26] and intramuscular fat infiltration was quantified by Oil Red O staining throughout the time course of regeneration (3, 7, and 14 days post-injury (dpi)). As expected, intramuscular fat infiltration peaked at 14 dpi in control mice. In contrast, *Pparg^Δ/Δ^* mice failed to induce ectopic adipogenesis as *Pparg^Δ/Δ^* muscles were completely devoid of any intra-muscular lipid accumulation (Figure 1A). The level of fat infiltration in *Pparg^Δ/Δ^* injured muscle was blunted by 98% (Figure 1B), and did not differ from non-injured contralateral muscles used as control. Thus, muscle ectopic adipogenesis, which develops transiently during regeneration, is due to bona fide adipocyte formation and fully relies on the presence of PPARγ.

To confirm the impaired adipogenic signature of *Pparg^Δ/Δ^* mice, we freshly isolated FAPs by flow cytometry and induced their differentiation into adipocytes ex vivo. As expected, approximately 45% of control FAPs differentiated in adipocytes after induction. In contrast, ex vivo adipogenesis was completely abolished in FAPs isolated from *Pparg^Δ/Δ^* muscles (reported hereafter as *Pparg^Δ/Δ^* FAPs) (Figure 1C,D). The loss of adipogenic fate of *Pparg^Δ/Δ^* FAPs did not primarily result from cell death as a large number of undifferentiated KO FAPs remained in culture after differentiation (Figure 1E) and *Pparg^Δ/Δ^* FAPs could efficiently expand and proliferate ex vivo. Nevertheless, we detected approximately 25% less *Pparg^Δ/Δ^* than control FAPs after differentiation (Figure 1E), likely because PPARγ is known to regulate pre-adipocyte clonal expansion, a process where adipogenic cells undergo a final cell division to exit the cell cycle and trigger adipogenic commitment and differentiation [43]. Altogether, our results confirmed the role of PPARγ as a master regulator of adipogenesis, and extended this observation to the context of intra-muscular adipogenesis. The complete absence of adipogenesis and ectopic fat infiltration in *Pparg^Δ/Δ^* muscle prompted us to use this model to investigate the influence of ectopic adipogenesis on muscle stem cell function, myogenesis, and muscle regeneration.

### 2.2. Loss of PPARγ Impairs Muscle Stem Cell Function and Cross-Talks with Myogenesis In Vivo

**Figure 2:**
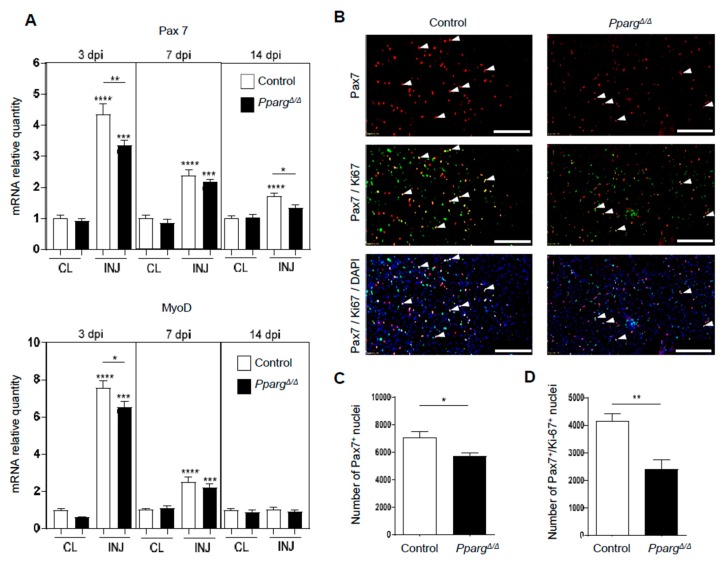
MuSC amplification and commitment are impaired in *Pparg^Δ/Δ^* mice. Tibialis anterior (TA) muscles from *Pparg^Δ/Δ^* and control littermates mice were injured with 50% glycerol intramuscular injection and collected at 3, 7, and 14 days post injury (dpi). (**A**) Pax7 and MoyD mRNA quantification by qPCR in injured (INJ) and non-injured contralateral (CL) muscles. Data are normalized to CL muscles of control mice per timepoint, *n* ≥ 7 mice per group. (**B**) Representative images of TA sections stained for Pax7, Ki-67, and DAPI at 7dpi. White arrows indicate Pax7^+^/Ki-67^+^ nuclei. Scale bar: 200 µm. (**C**,**D**) In vivo quantification of total Pax7^+^ nuclei (**C**) and proliferating Pax7^+^/Ki-67^+^ nuclei (**D**) in injured muscles at 7dpi, with *n* = 4–5 mice per group. **** *p* < 0.0001, *** *p =* 0.001, ** *p* = 0.01, * *p* < 0.05 by one-way ANOVA (**A**) or Mann–Whitney test (**C**,**D**). Unless otherwise indicated, statistical comparisons relate to the CL muscle of control mice.

To interrogate whether MuSC function and muscle repair are affected in *Pparg^Δ/Δ^* mice, we first quantified the expression of Pax7 and MyoD, which are expressed by regenerating cells, during the time course of muscle repair. As expected, we observed a strong upregulation of Pax7 and MyoD at 3 dpi, as a consequence of MuSC activation in response to injury (Figure 2A). Interestingly, the induction of Pax7 and MyoD was impaired in *Pparg^Δ/Δ^* mice during the time course of regeneration. Similar analyses in contra-lateral (CL) muscles demonstrated that these myogenic markers were not deregulated in non-injured *Pparg^Δ/Δ^* muscles, demonstrating that lack of PPARγ specifically impairs the myogenic activity of MuSCs after their activation in response to injury. In order to confirm the evidence obtained at a molecular level, we next analyzed the in vivo activity of MuSCs by immunofluorescence against Pax7. The total number of Pax7-positive MuSCs was lower in *Pparg^Δ/Δ^* than control muscle after injury (Figure 2B,C), confirming that *Pparg^Δ/Δ^* mice fail to efficiently amplify myogenic precursors during muscle regeneration. Altered amplification of *Pparg^Δ/Δ^* MuSCs resulted from impaired proliferation of MuSCs as the number of Pax7^+^/Ki-67^+^ proliferating MuSCs was significantly reduced by approximately 45% in *Pparg^Δ/Δ^* muscle (Figure 2B–D).

**Figure 3:**
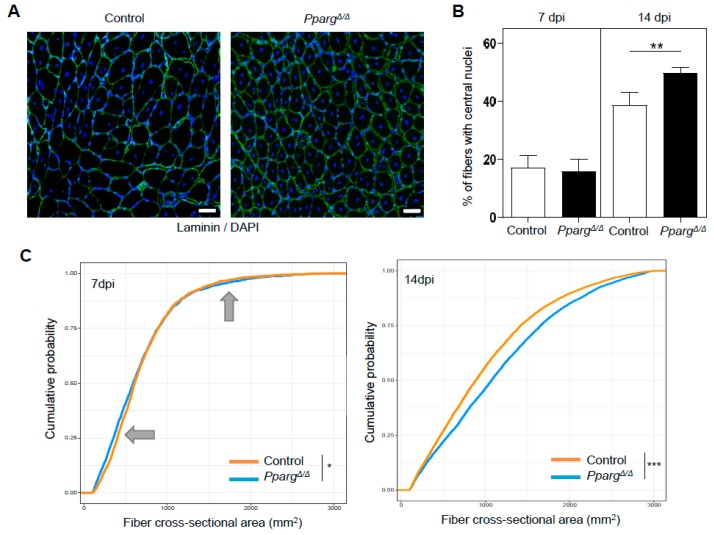
Muscle regeneration is impaired in *Pparg^Δ/Δ^* mice. (**A**) Representative images of *Pparg^Δ/Δ^* and control muscle sections stained with Laminin and DAPI at 14dpi. Scale bar: 50 µm. (**B**) Quantification of the percentage of fibers possessing centralized nuclei at 7 and 14dpi, with n=7 mice per group. (**C**) Cumulative distribution of cross sectional area of regenerating fibers with centralized nuclei in regenerating muscles of *Pparg^Δ/Δ^* and control littermates mice at 7dpi (left panel) and 14dpi (right panel). Gray arrows depict the bimodal differences of *Pparg^Δ/Δ^* and control cross sectional area distributions. *n* = 7 mice per group. *** *p* = 0.001, ** *p* = 0.01, * *p* < 0.05 by Mann–Whitney test (**B**) or Kolmogorov–Smirnov test (**C**).

To investigate how the MuSC phenotype of *Pparg^Δ/Δ^* mice influences myogenesis and muscle repair, we quantified the amount of regenerating fibers with centralized nuclei as well as well their cross-sectional area during the time course of muscle regeneration. Loss of PPARγ did not affect the size of the injured area as the percentage of fibers with centralized nuclei was equal in control and *Pparg^Δ/Δ^* mice during the early steps of regeneration (Figure 3B). However, the muscle of *Pparg^Δ/Δ^* mice failed to efficiently regenerate as they had more fibers with centralized nuclei at late stages of regeneration (Figure 3A,B), indicating that their recovery to fully matured fibers with peripherally located nuclei was delayed. At 7dpi when MuSCs start to fuse with damaged or newly formed fibers, fiber size distribution was significantly impaired in *Pparg^Δ/Δ^* mice in a bimodal fashion (Figure 3C; gray arrows). The smallest fibers, which encompass the newly formed fibers through de novo MuSC fusion, were smaller in *Pparg^Δ/Δ^* mice, indicating that the defective MuSC amplification in *Pparg^Δ/Δ^* mice translates to perturbed myogenesis and altered MuSC fusion. The largest fibers, which likely correspond to the repair of pre-existing fibers that were only mildly damaged were larger in *Pparg^Δ/Δ^* mice and this phenotype persisted at later time points of regeneration (Figure 3C), likely indicating that PPARγ can influence myofiber growth through additional mechanisms which are MuSC-independent. Altogether, these results demonstrate that loss of PPARγ impairs MuSC expansion and myogenesis in vivo and cross-talks with muscle repair.

### 2.3. PPARγ KO Does Not Directly Impair the Intrinsic Function of MuSC Function and the FAP/MuSC Cross-Talk Ex Vivo

**Figure 4:**
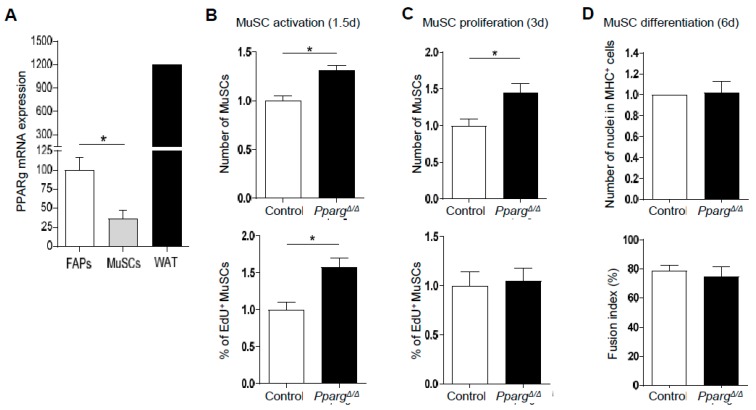
*Pparg^Δ/Δ^* MuSCs activate faster than control MuSCs ex vivo. (**A**) PPARγ mRNA quantified by qPCR in freshly isolated MuSCs, FAPs and mouse white adipose tissue (WAT). *n* = 4 mice (**B**,**C**) Relative number (top panel) and percentage of EdU^+^ (lower panel) MuSCs cultured for 36 h (**B**) or 72 h (**C**) after FACS isolation. (**D**) Myogenic differentiation of *Pparg^Δ/Δ^* and control MuSCs cultured for six days after FACS isolation. Relative quantification of the total number of nuclei having fused in MHC + myofibers is shown in the top panel, and fusion index (% nuclei in MHC + myofibers) is shown in the lower panel. (**B**,**D**) *n* ≥ 24 cell culture replicates pooled from at least three mice per genotype and replicated twice on different days. * *p* < 0.05; by Mann–Whitney test.

Since muscle regeneration is regulated by an integrated cooperation between many residing and recruited cell types in the MuSC niche, we asked whether the in vivo MuSC phenotype of *Pparg^Δ/Δ^* mice is either due to a direct intrinsic role of PPARγ in MuSCs, or indirectly influenced by a PPARγ-dependent paracrine signal from other cell types of the MuSC niche. As a first step, PPARγ expression in freshly isolated MuSCs and FAPs was compared to its expression in white adipose tissue (Figure 4A). PPARγ expression was detected in MuSCs, but at lower levels than in FAPs and much lower than in adipose tissue (Figure 4A), thus making it unlikely that PPARγ could drive MuSC fate in a cell-autonomous fashion. This was confirmed by demonstrating that the lower activation and proliferation of MuSCs detected in vivo in *Pparg^Δ/Δ^* mice was not reproduced in an ex vivo assay with Edu incorporation 1.5 and 3 days after MuSCs isolation from control and *Pparg^Δ/Δ^* mice (Figure 4B,C). Surprisingly, the ex vivo activation during the first cellular division after exit of quiescence was even greater in *Pparg^Δ/Δ^* cells than in control cells (Figure 4B). Given the low expression of PPARγ in MuSCs and the fact that the proliferation of *Pparg^Δ/Δ^* MuSCs was normal and steady after 3 days of culture, higher ex vivo activation of *Pparg^Δ/Δ^* MuSCs is likely caused by indirect mechanisms. This was further confirmed by assessing the terminal differentiation of *Pparg^Δ/Δ^* MuSCs into myofibers after 6 days of ex vivo culture. Neither the number of differentiated myosin-heavy chain (MHC)-positive cells nor the fusion index of nuclei in multi-nucleated myofibers differed between *Pparg^Δ/Δ^* and control MuSCs (Figure 4D).

**Figure 5:**
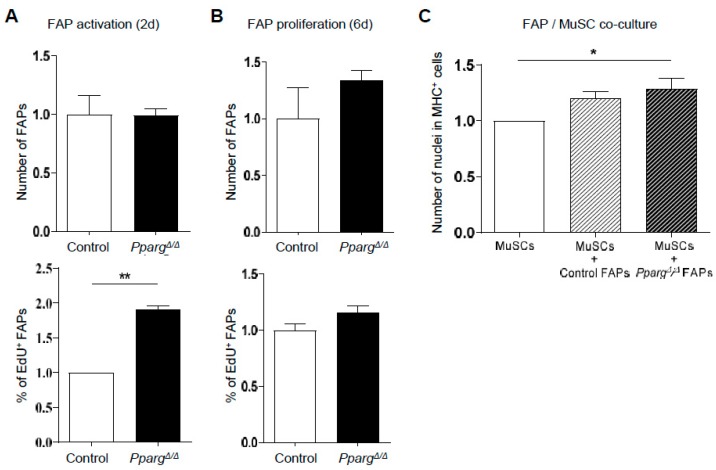
PPARγ deficiency does not affect the myogenic support of undifferentiated FAPs to MuSCs. (**A**,**B**) Relative number (top panels) and percentage of Edu-positive FAPs from *Pparg^Δ/Δ^* and control littermates mice cultured for 48 h (**A**) and six days after isolation (**B**,**C**) Relative number of differentiated MHC^+^ MuSCs, when cultured alone or co-cultured with *Pparg^Δ/Δ^* and control FAPs. Data are normalized to MuSCs monoculture. *n* ≥ 24 cell culture replicates pooled from at least three mice per genotype and replicated twice on different days ** *p* < 0.01, * *p* < 0.05 by Mann–Whitney test.

We then tested whether PPARγ could indirectly regulate MuSC function by regulating the cellular cross-talk between MuSCs and other PPARγ-expressing accessory cells in the muscle stem cell niche. FAPs appeared as good candidates as they support MuSC activity as progenitors [19] and possess an adipogenic fate. We first evaluated how loss of PPARγ influences FAP expansion ex vivo using total cell counts and EdU incorporation to reveal activated and proliferating cells 2 and 6 days after isolation, respectively. After 2 days in culture, we did not detect any difference in the total FAP number, highlighting that both control and *Pparg^Δ/Δ^* FAPs had similar adhesion and survival (Figure 5A). Cell cycle entry assessed by Edu incorporation was faster in *Pparg^Δ/Δ^* FAPs 2 days post-isolation (Figure 5A), but did not result in more FAPs or higher proliferation 6 days post-isolation (Figure 5B). Thus, while PPARγ is essential for FAP differentiation to adipocytes, it does not play an intrinsic role in the regulation of FAP expansion. We next interrogated the role of PPARγ in the cross talk between FAPs and MuSCs through direct ex vivo co-cultures. As expected, co-culture of MuSCs with control FAPs was beneficial for their myogenic differentiation in myosin-heavy chain positive myotubes (Figure 5C). However, *Pparg^Δ/Δ^* FAPs also supported myogenic differentiation of MuSCs to a similar extent (Figure 5C). These results therefore revealed that in their undifferentiated precursor state, FAPs efficiently sustain the myogenic differentiation of MuSCs through PPARγ-independent mechanisms.

### 2.4. Altered Inflammatory Signatures in PPARγ-KO Mice During Muscle Regeneration

**Figure 6:**
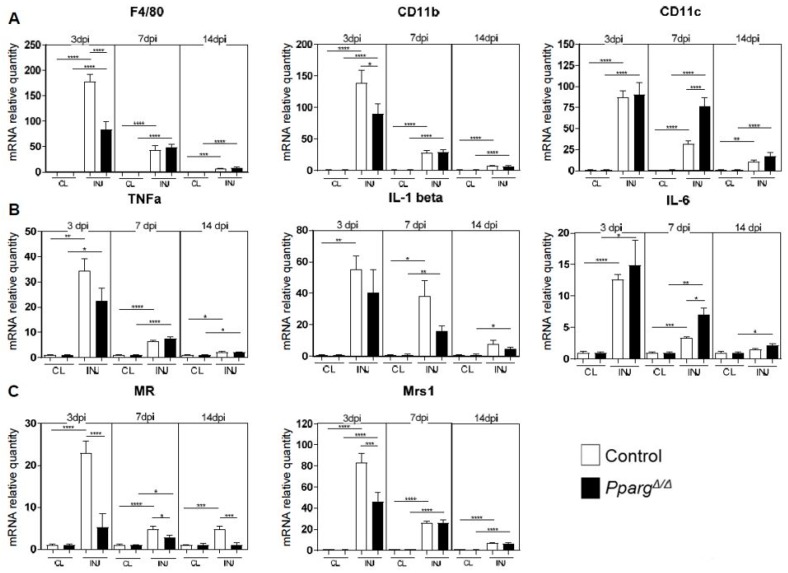
The inflammatory response during muscle regeneration is altered in *Pparg^Δ/Δ^* mice. mRNA quantification by qPCR of immune cell marker in injured (INJ) and non-injured contra-lateral (CL) muscles at 3, 7 and 14 dpi. Results are normalized to the non-injured CL muscles. (**A**) Cellular surface macrophages markers: *F4/80 = EMR1* (EGF-like module-containing mucin-like hormone receptor-like 1), *CD11b* = Integrin alpha M, *CD11c* = Integrin alpha X. (**B**) Cytokines: *TNFα =* Tumor necrosis factor alpha, *IL-1 β* = Interleukin 1 beta, *IL-6* = Interleukin 6. (**C**) Anti-inflammatory cellular macrophages markers: *MR* = mannose receptor, *Msr1* = Macrophage scavenger receptor 1. *n* ≥ 7 mice. **** *p* < 0.0001, *** *p* < 0.001, ** *p* < 0.01 * *p* < 0.05 by one-way analysis of variance (ANOVA). Unless otherwise indicated, statistical comparisons relate to the CL condition of the same genotype.

Our results demonstrating that the intrinsic loss of PPARγ in MuSCs and FAPs does not directly cause the MuSC activation and regeneration defects of *Pparg^Δ/Δ^* mice prompted us to evaluate for a role of PPARγ in other cell types that remodel the stem cell niche during regeneration. In particular, the fact that MuSCs are normal in uninjured muscle of *Pparg^Δ/Δ^* mice and only affected upon muscle regeneration in a dynamic fashion during the early steps of muscle repair suggested a potential role of PPARγ in a cell type recruited to damaged muscle after injury. We thus quantified the expression level of macrophage surface markers and chemokines in control and *Pparg^Δ/Δ^* mice. General inflammatory markers such as F4/80, CD11b, and CD11c were differentially regulated in injured muscles of *Pparg^Δ/Δ^ vs* control mice at various time points (Figure 6A), while the induction of IL-1, TNF and IL-6 mRNA in response to injury did not change significantly at 3dpi between *Pparg^Δ/Δ^* and control muscles (Figure 6B). The analysis of anti-inflammatory macrophage cell surface markers revealed a defect in regenerating muscle of *Pparg^Δ/Δ^* mice, as deletion of PPARγ severely blunted the induction of the mannose receptor (MR) and the macrophage scavenger receptor (MRS1) at 3dpi (Figure 6C), when the regenerating niche shifts to anti-inflammatory conditions to support myogenic commitment. Resolution of the IL-6 inflammatory cytokine production at 7dpi was also altered in *Pparg^Δ/Δ^* (Figure 6B). These observations highlight that PPARγ influences the temporal profiles of the inflammatory response during muscle regeneration. Altogether, our study demonstrates that whole body loss of PPARγ influences muscle regeneration by acting on MuSC function indirectly through various complementary mechanisms in various cell types of the stem cell niche.

## 3. Discussion

Ectopic infiltration of adipocytes in skeletal muscle has been widely associated with altered muscle function, mainly because adipose infiltration is observed in many muscle degenerative conditions such as dystrophies or rotator cuff tears [9,10,11,12]. In contrast, recent studies have suggested that muscle adipogenesis may actually be a physiological process required for muscle plasticity, which gets deregulated and over-amplified during pathology. First, ectopic muscle adipogenesis is detected in several models of efficient muscle regeneration [24,25,26,27], and this response is transient and cleared during the terminal phases of muscle repair when myofibers return to homeostasis. In addition, the FAP lineage can both support myogenesis and differentiate to intra-muscular adipocytes, with different states of permissivity according to the patho-physiological status [19,20]. Finally, muscle adipogenesis is blunted in response to unloading and lack of muscle contraction [25]. In the present study, we have demonstrated that the ectopic accumulation of fat during muscle regeneration fully relies on the adipogenic transcription factor PPARγ. *Pparg^Δ/Δ^* mice are totally devoid of intra-muscular adipocytes after muscle injury and *Pparg^Δ/Δ^* FAPs completely lose the ability to differentiate to adipocytes. On top of confirming the dominant role of PPARγ on adipogenesis [40] in skeletal muscle, this result also highlights that muscle ectopic fat formation after injury is an active cellular process tightly regulated at the molecular level, and not just the aspecific accumulation of cellular lipids from damaged myofibers.

Interestingly, our results consistently demonstrate that the absence of PPARγ and ectopic adipocyte formation alter the activation and myogenic commitment of MuSCs in vivo. The induction of Pax7 and MyoD as well as the proliferation of Pax7-positive MuSCs are impaired in the regenerating muscle of *Pparg^Δ/Δ^* mice. PPARβ/δ another member of the PPAR nuclear receptor family, has previously been demonstrated to control MuSC function and muscle regeneration [44,45,46], most likely through a direct effect on the myogenic lineage as Myf5-Cre conditional deletion of PPARγ is sufficient to alter MuSCs and regeneration [44]. In contrast, the alterations of muscle regeneration in *Pparg^Δ/Δ^* mice in vivo did not result from cell autonomous defects linked to the absence of PPARγ in MuSCs as PPARγ expression is low in MuSCs and the activation and proliferation of *Pparg^Δ/Δ^* MuSCs was not impaired ex vivo. Thus, the perturbed regenerative response of *Pparg^Δ/Δ^* mice support the notion that a functional adipogenic response is required to efficiently sustain myogenesis during physiological muscle healing. In particular, we can hypothesize that cellular and membrane lipids from damaged myofibers are released in the muscle stem niche a few days after injury and need to be stored intracellularly to avoid damage to regenerating fibers. Interestingly, the adipogenic lineage has also been demonstrated to support self-renewal and regeneration in other organs. For example, adipocyte precursors have been involved in skin epithelial stem cell activation in the hair follicle [47,48], and the adipogenic lineage was shown to recruit fibroblasts during skin wound healing [49]. A cross-talk between adipose-derived stem cells and chondrocytes has also recently been found in vitro, potentially revealing similar cross-communications during cartilage repair [50].

Our study has analyzed muscle regeneration in a mouse model where PPARγ is deleted in all cells and organs [39,40]. At the undifferentiated progenitor level, we demonstrated that control and *Pparg^Δ/Δ^* FAPs had a similar ability to support myogenesis. We also further showed that the function of isolated *Pparg^Δ/Δ^* MuSCs is not lower ex vivo. Surprisingly, *Pparg^Δ/Δ^* MuSCs even activated faster than control cells ex vivo, possibly because the lipodystrophy and metabolic phenotype of *Pparg^Δ/Δ^* mice alter MuSC quiescence in vivo and trigger earlier activation ex vivo. Given these observations, it is therefore likely that impaired in vivo MuSC function in *Pparg^Δ/Δ^* mice is indirect, through a cross-talk with other cell types of the muscle stem cell niche or via systemic signals from other tissues. Along this line, two mechanisms most likely cross-talk during regeneration. First, the absence of adipogenic differentiation of *Pparg^Δ/Δ^* FAPs can contribute to the in vivo phenotype by controlling adipokine signals during adipogenic and myogenic differentiation. Second, the immune signature is altered in the regenerating niche of *Pparg^Δ/Δ^* mice, where anti-inflammatory macrophage markers fail to get efficiently induced. Recent work has demonstrated that PPARγ in macrophages regulates myogenesis and muscle repair via secreted paracrine signals [33]. Our results are consistent with the indirect modulation of myogenesis via PPARγ in immune cells and fully supports the model where macrophage PPARγ cross-talks with MuSCs through secreted cytokines. Interestingly, the fate of FAPs is tightly controlled by inflammatory cytokines, which dynamically influence their expansion, differentiation and apoptosis during the resolution of inflammation and the different phases of muscle repair [23,51,52,53]. Thus, PPARγ most likely influences MuSCs and myogenesis indirectly via a complex cross-talk between macrophages and FAPs.

Altogether, our work has demonstrated that PPARγ is required for ectopic adipocyte infiltration during muscle regeneration and alters MuSC proliferation and myogenic commitment via indirect cellular cross-talks in the stem cell niche. Genetic PPARγ invalidation in macrophages and bone marrow transplant from PPARγ KO mice have already demonstrated that macrophages are required for this cross-talk [33]. Further studies with cell-type specific invalidation of PPARγ, in particular in FAPs, will be important to further refine the roles of the distinct cellular compartments of the niche and the interactions between adipogenesis and myogenesis during muscle regeneration and muscle diseases.

## 4. Materials and Methods

### 4.1. Animals

Animals were housed under standard conditions and allowed to access to food and water ad libitum. Since the whole-body *Pparg* null mice die during embryonic development as a result of placental defects, we used mice obtained through an epiblast-specific Cre recombinase expression (*Sox2-Cre*^tg/+^:*Pparg^Δ/^*^em*Δ*^ mice, hereafter called *Pparg^Δ/Δ^* mice) as it has been demonstrated that PPARγ is necessary for placental function but not for embryo development [39]. The generation and breeding of mice for these experiments was performed as previously described [40]. Briefly, *Sox2-Cre*^tg/+^ male mice (Jackson Laboratory, Bar Harbor, ME, USA) were mated with female *Pparg^Δ/+^* mice [54] to obtain male *Sox2-Cre*^tg/+^:*Pparg^Δ/+^* mice that were next mated with *Pparg^fl/fl^* females to obtain *Sox2-Cre*^tg/+^:*Pparg^Δ/^*^em*Δ*^ mice (*Pparg^Δ/Δ^*) and *Sox2-Cre*^+/+^:*Pparg^fl/^*^+^ (control). All experimental animals were breeding littermates of a mixed C57BL6/SV129 genetic background, and control animals were PPARg^fl/+^ that do not express Sox2^Cre^. Only female mice were used in this study since adult male *Pparg^Δ/Δ^* mice present a higher mortality. For muscle regeneration experiments, 11–15 week old mice were anaesthetized with 2% isoflurane and 50 uL of 50% v/v glycerol was injected into the tibialis anterior (TA) muscle. Mice received buprenorphine (Temgesic) 0.1 mg/kd/day i.p. as analgesic during the 48h following glycerol injection. Mice were sacrificed by CO_2_ inhalation followed by cervical dislocation 3, 7, 14 days post-injury (dpi), and regenerating and contra-lateral TA muscles were cut in half and snap-frozen in liquid nitrogen for molecular analyses or frozen in liquid nitrogen cooled isopentane for histology. All in vivo experiments were performed following the regulations of the Swiss Animal Experimentation Ordinance and approved by the ethical committee of the canton de Vaud under license VD2818, 01 Mar 2014.

### 4.2. Muscle Progenitor Cell Isolation

For the isolation of muscle stem cells, uninjured hindlimb muscles representing various fiber types (gastrocnemius/soleus/plantaris complex, tibialis anterior/EDL complex and quadriceps) were collected and from *Pparg^Δ/Δ^* mice and their control littermates and freshly digested with dispase II (2.5 U/mL) (Roche, Basel, Switzerland), collagenase B (0.2%) (Roche), and MgCl_2_ (5 mM). Cells were then incubated at 4 °C for 30 min with fluorescently-coupled antibodies against CD45 (Invitrogen, Carlsbad, CA, USA) MCD4501 or MCD4528; dilution for both 1/25), CD31 (Invitrogen, RM5201 or RM5228; dilution for both 1/25), CD11b (Invitrogen, RM2801 or RM2828; dilution for both 1/25), CD34 (BD Biosciences, 560230 or 560238; dilution for both 1/60), Ly-6A–Ly-6E (Sca1) (BD Biosciences, Franklin Lakes, NJ, USA) 561021; dilution 1/150), α7-integrin (R&D, FAB3518N; dilution 1/30) and CD140a (eBioscience, San Diego, CA, USA, 12-1401-81 or 17-1401-81; dilution for both 1/30). Cell isolation was performed on a Beckman–Coulter Astrios Cell sorter; Beckman-Coulter, Brea, CA, USA. MuSCs isolated by flow-cytometry were CD45^−^CD31^−^CD11b^−^Sca1^−^CD34^+^Integrinα7^+^; fibro/adipogenic progenitors (FAPs) were CD45^−^CD31^−^CD11b^−^Sca1^+^CD34^+^PDGFRα^+^.

### 4.3. Cell Culture

MuSCs and FAPs were sorted in 96 well plates at a density of 600 cells per well. Freshly sorted cells were grown in growth medium: 20 mM glucose DMEM, 20% heat-inactivated FBS, 10% inactivated horse serum, 2.5 ng/mL bFGF (Invitrogen), 1% P/S + 1% L+-Glutamine, 1% Na-pyruvate (Invitrogen). For MuSC activation 1µM EdU (5-Ethynyl-2′-deoxyuridine) was added to the growth medium after cell sorting. Cells were then fixed 1.5 days after with 4% paraformaldehyde (PFA) for 15 min. For MuSC proliferation, 1 µM EdU was added to the growth medium 3 days after cell sorting, for 2.5 h. Cells were then fixed 15 min in 4% PFA. MuSC differentiation was induced after 4 days in culture by switching to myogenic differentiation medium (20 mM glucose DMEM, 5% inactivated horse serum, 1% P/S). Cells were cultured in these conditions for two days and then fixed in 4% PFA for 5 min. For FAP activation 1 µM EdU was added to the growth medium after cell sorting. Cells were then fixed two days after with 4% PFA for 15 min. For FAP proliferation, 1 µM EdU was added six days after cell sorting. Cells were then incubated for 6 h and fixed in 4% PFA for 15 min. For FAP differentiation, after six days of culture in growth medium, the medium was replaced by adipogenic differentiation medium for an additional seven days (20 mM glucose DMEM, 20% heat-inactivated FBS, 1% P/S, 0.25 µM dexamethasone, 1 µg/mL insulin, 5 µM troglitazone, 0.5 mM isobutylmethylxanthine). For MuCS and FAP co-cultures, we cultured PPARγ-KO MuSCs alone, or with WT or PPARγ-KO FAPs.

### 4.4. Immunocytochemistry for In Vitro Studies

EdU incorporation was revealed by the Click-iT assay (Molecular Probes, Eugene, OR, USA) according to manufacturer’s instructions. After fixation, cells were permeabilized during 20 min in PBTX 0.5%, stained with the Click-iT reaction mix, and counterstained with DAPI. For myosin heavy vhain (MHC) staining, after fixation, cells were permeabilized using EtOH/MetOH (*v/v*) for 5 min, incubated for 1 h with the primary antibody anti-MHC 1/200 (Millipore clone A4.1025) in PBS, 1% Horse Serum at room temperature, and incubated during 30 min with the secondary antibody Alexa488 anti-mouse IgG diluted at 1/1000 (Life Tech (Carlsbad, CA, USA) A-10680) and Hoeschst33342 in PBS, 1% horse serum at room temperature. Fusion index was determined as the percentage of nuclei located within MHC positive fibers. Image acquisition was performed using the ImageXpress (Molecular Devices, San Jose, CA, USA) platform. Quantifications were done using the MetaXpress software (Molecular Devices).

### 4.5. Immunohistochemistry for In Vivo Studies

TA muscles were frozen in isopentane and cooled with liquid nitrogen. Sections of 10 μm were obtained after sectioning on a cryostat. For Pax7-Ki67 staining, sections were allowed to dry for 10 min and then fixed 10 min in 4% PFA. Tissue sections were permeabilized by applying cold methanol (−20 °C) for 6 min. For the antigen revealing, slides were immersed in a glass container filled with hot acid citric (0.01 M), and then in boiling water bath for 5 min, repeated twice. Slides were then blocked 3 h in blocking solution (IgG-free Bovine Serum Albumin (BSA) 4% (Jackson #001-000-162), followed by 30 min incubation with Goat-anti-mouse FAB (Jackson #115-007-003) diluted 1/50 in PBS. Rabbit anti-Ki67 polyclonal (Abcam, Cambridge, United Kingdom, #ab15580) was used (1/200) and chicken anti-laminin (Life-Span Bioscience, Seattle, WA, USA, #LS-C96142) antibodies were incubated 3 h in blocking solution in1/200 and 1/300 dilution, respectively. Mouse anti-Pax7 (DSHB, purified) IgG1 (2.5 µg/mL) was then incubated overnight at 4 °C. Secondary antibodies (Alexa488-Goat anti-rabbit (1/1000) and Alexa647-Goat anti-chicken (1/1000) were incubated for 1 h in blocking solution. Pax7 signal was further amplified using a goat-anti mouse IgM1-biotin (Jackson), followed by a streptavidin (1/2000) treatment, together with Hoechst.

For Oil-Red-O staining slides were air-dried and then incubated in 50% ethanol during 30 min, followed by 15 min incubation in 2.5 g/L oil-red-O solution in 70% ethanol and 1 min washes in 50% ethanol and water. Finally, slides were counterstained with Mayer’s hematoxylin. Stained tissues were imaged using an Olympus VS120 virtual microscopy slide scanning system and analyzed using the VS-ASW FL software measurement tools. The Pax7-Ki67 double positive nuclei (DAPI positive) were counted by randomly selecting four or more areas within the injured region. Fiber size and Oil-Red-O area analyses were performed on an automatized software developed in house.

For BODIPY staining, BODIPY 493/503 (4,4-difluro-1,3,5,7-tetramethyl-4-bora-3a,4a-diaza-s-indacene, Thermo Fisher Scientific, Waltham, MA, USA) was used according to manufacturer’s instructions. Images were acquired with a Leica microscope (DMI4000B Leica widefield inverted microscope; Leica, Wetzlar, Germany), at 20× magnification.

### 4.6. Quantitative PCR

RNA was extracted from sorted cells or muscles and white adipose tissue using RNeasy Micro Kit (Qiagen, Hilden, Germany) or miRNeasy Mini Kit (Qiagen), respectively. According to the manufacturer’s instruction, cells were stored in RTL buffer and frozen after the FACS sorting. RNA samples were processed by reverse transcription (high capacity cDNA reverse transcription kits, Invitrogen) using random primers (High Capacity cDNA Reverse Transcription Kit, Applied Biosystems, Foster City, CA, USA. All of the quantitative PCRs were performed using the SybR Green I master kit (Roche) on a LightCycler 480. For sorted cells and adipose tissue, reference gene HPRT was selected. PPARγ primers were designed on the gene region deleted in the KO mice:

Forward: AAGAGCTGACCCAATGGTTG, reverse: GCATCCTTCACAAGCATGAA. For muscles, three reference genes (ATP5b, EIF2a, and PSMB4) were selected from previous micro-array data on the base of their stability through the different time points of regeneration.

qPCR probes were designed as follows:

**ATP5b** forward: ACCTCGGTGCAGGCTATCTA

**ATP5b** reverse: AATAGCCCGGGACAACACAG;

**EIF2a** forward: CACGGTGCTTCCCAGAGAAT

**EIF2a** reverse: TGCAGTAGTCCCTTGTTAGCG;

**PSMB4** forward: GCGAGTCAACGACAGCACTA

**PSMB4** reverse: TCATCAATCACCATCTGGCCG;

**Pax7** forward: AAGTTCGGGAAGAAAGAGGACGAC

**Pax7** reverse: GAGGTCGGGTTCTGATTCCACATC;

**MyoD** forward: GCAGATGCACCACCAGAGTC

**MyoD** reverse: GCACCTGATAAATCGCATTGG;

**F4/80** forward: CTCTTCTGGGGCTTCAGTGG

**F4/80** reverse: TGTCAGTGCAGGTGGCATAA;

**ITGAM (CD11b)** forward: GCCTGTGAAGTACGCCATCT

**ITGAM (CD11b)** reverse: GCCCAGGTTGTTGAACTGGT;

**ITGAX (CD11c)** forward: ACACTGAGTGATGCCACTGT

**ITGAX (CD11c)** reverse: TTCGGAGGTCACCTAGTTGGG;

**Msr1** forward and reverse: [24]

**MR** forward: ATGCCAAGTGGGAAAATCTG

**MR** reverse: TGTAGCAGTGGCCTGCATAG;

**TNFα** forward: AGCCGATGGGTTGTACCTTG

**TNFα** reverse: ATAGCAAATCGGCTGACGGT;

**IL1beta** forward: TGCCACCTTTTGACAGTGAATGA

**IL1beta** reverse: TGCCTGCCTGAAGCTCTTGT;

**IL-6** forward: CCAGAAACCGCTATGAAGTTCC

**IL-6** reverse: TTGTCACCAGCATCAGTCCC;

### 4.7. Statistical Analysis

In vitro experiments were performed in at least three independent replicates from different mice, using several cell culture replicates from the same isolation. All of the data are expressed as mean value + the standard error of the mean (s.e.m.). For statistical analysis, GraphPad Prism (GraphPad Software Inc., La Jolla, CA, USA) was used. Statistical significance of two-group comparisons was assessed by a non-parametric Mann–Whitney test which does not assume normal distribution, using variance correction when required. For comparison of more than two groups, one-way ANOVA followed by Bonferroni multiple-comparison test was used. 

## Supplementary Materials

Supplementary materials can be found at http://www.mdpi.com/1422-0067/19/7/2044/s1.
