# Supplementary figures and images for: PPARγ Controls Ectopic Adipogenesis and Cross-Talks with Myogenesis During Skeletal Muscle Regeneration
Source: Int J Mol Sci. 2018 Jul 13;19(7):2044. doi: 10.3390/ijms19072044 (PMC6073847; doi:10.3390/ijms19072044)

FIGURE S1

A

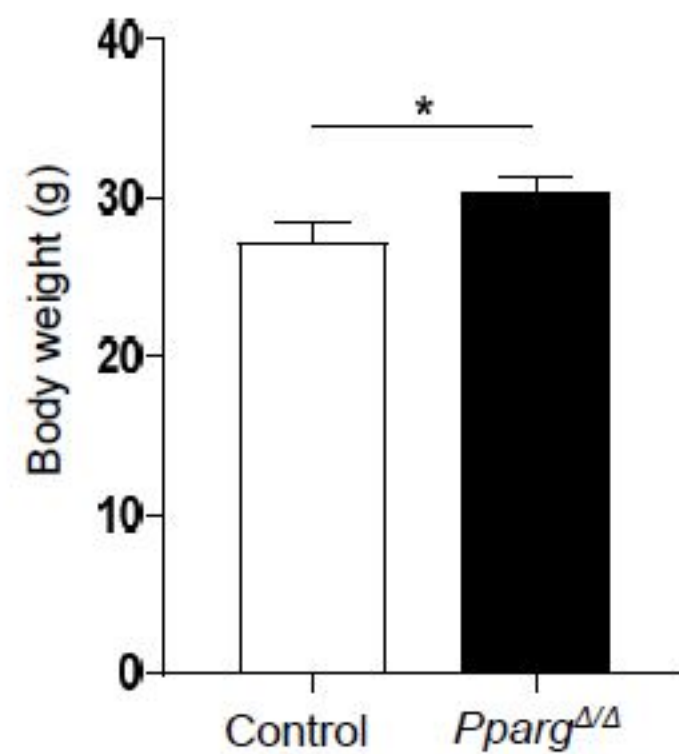

B

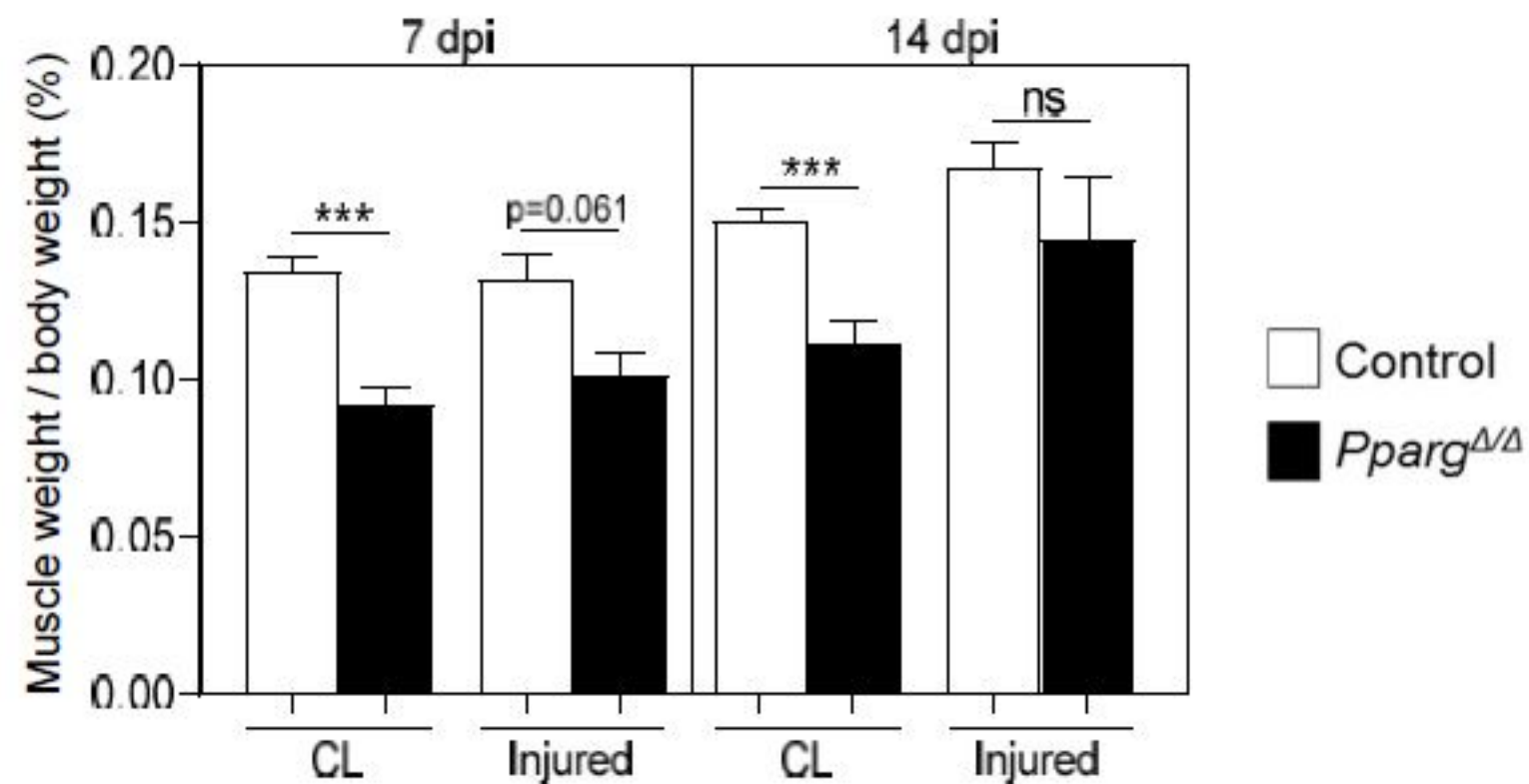

Supplement: Supplementary file 1 [file ijms-19-02044-s001.pdf]
